# Virus against virus (VIVI): a potential solution against HIV/AIDS

**DOI:** 10.1186/1755-7682-7-19

**Published:** 2014-05-06

**Authors:** Asfandyar Sheikh, Muhammad Farhan Khaliq, Muhammad Muslim Noorani

**Affiliations:** 1 Dow Medical College, Dow University of Health Sciences, Karachi, Pakistan

**Keywords:** HIV, AIDS, GBV-C, Hepatitis G virus, Super-infection, HAART, Vaccination

## Abstract

Most therapeutic regimens are aimed at the use of pharmacologic agents or the induction of immunological response against the pathological agent. However, these methods tend to be insufficient for the management of some of the most debilitating infectious diseases. Here we present a novel therapeutic approach. It involves voluntary super-infection of a subject having HIV/AIDS with a virus (GBV-C), which to date has not been shown to be responsible for any pathology. It has been shown to counter, suppress or eradicate the agent responsible for the severe disease. Several studies demonstrate the role of different micro-organisms in influencing the growth of other pathogens in the human body. This hypothesis requires meticulous testing before its implementation on humans. If the trials are successful, the implications for this hypothesis are promising considering the compliance issues and adverse effects associated with current standard of HIV care.

## Background

A number of micro-organisms, specially bacteria and fungi are present as part of human normal flora, as our normal body homeostasis provides a dynamic medium to develop a symbiotic relationship. These organisms are present in areas like nasal cavity, gastrointestinal tract, respiratory tract, genital tract etc. serving as “ecological niches” and strengthen the immunity by restricting the proliferation of pathogenic organisms. However, disturbed homeostasis, for example, by injudicious use of antibiotics or immunosuppression, allows uncontrolled growth of these organisms, resulting in pathogenicity.

Humans exhibit immunological diversity as suggested by the fact that different diseases follow a different course of illness and resolution in different individuals. This depends upon host factors such as genetic makeup and immunological response to an attack along with the capacity of pathogen viral genome to attack. What remains under-examined is the role of potentially beneficial organisms which have the capacity to alter the course of illness by interacting with the body and its other normal residents or the new invaders.

Important advancements have been made in medicine by observing the change in profile of symptoms of one disease caused by another disease process. An example is Edward Jenner’s efforts in prevention of smallpox by using cowpox pustular lesions. Following in Jenner’s footsteps, we propose eradication, or at least suppression of existing infection with infection from another biological agent which is not pathogenic, or with attenuated pathogenicity.

## Presentation of the hypothesis

In the domain of clinical medicine, interventions for promotion of good health can be divided into preventive medicine and therapeutic medicine. The former deals with modalities that are specially designed to prevent or delay the onset of disease processes (an example is vaccinations), the latter is concerned with the treatment of pathological conditions resulting once the disease process has initiated (an example is genetic therapy using adenovirus for cystic fibrosis patients). Thus, the use of micro-organisms which are not pathologic is well-documented in medicine.

We propose a novel treatment method for patients having AIDS disease. It involves voluntary super-infection of such subjects with GB virus C (GBV-C; formerly called as Hepatitis G virus).

This proposed treatment is based on findings of several randomized control trials which document a change in the progression of an infectious disease due to concomitant infection with a different infectious agent [1-4]. The introduced agent has been shown to result in the remission of the previous infection or at least a decrease in viral load and disease progression.

Hepatitis G (GBV-C) is an enveloped, single stranded RNA virus belonging to family Flaviviridae having close relationship with Hepatitis C sharing approximately 30% amino acid homology [5]. It is transmitted through the same modes HIV uses to transmit itself to its host. To date, there is no evidence of any known pathogenicity of GB Virus C in humans and most of its carriers have not shown any symptomatic infection like those infected by other members of the same family [6-8]. Although it is rare to find beneficial effects of viruses, in vitro studies have reported an inhibitory role of Flavi Family (GBV-C, DV, hepatitis C virus, West Nile virus, and yellow fever virus) on CD4+ T cells against HIV [9].

In Western countries, up to 4% of healthy blood donors are GBV-C viremic, while in developing countries the prevalence rates can be as high as 20% [10-14]. And those who are at risk of transmission of other infections such as HIV, HBV and HCV have prevalence rates approaching 50% [13,15,16].

Since the mode of transmission is same, GBV-C infection is common in people with HIV infection and is surprisingly associated with significantly improved survival, while decrease in GBV-C viral loads are linked to HIV progression and increased mortality [1,17]. Similar findings have been reported by other studies [18,19]. Although in the study conducted by Vahidnia et al., there was incidental exposure suggesting improved survival in HIV patients on highly active anti-retroviral (HAART) therapy; a randomized control trial will provide us a more accurate answer whether such association is actual or spurious. Another study reported decreased vertical transmission of HIV in infants positive for GBV-C RNA [20]. Thus all these studies point towards a possible role of GBV-C in the suppression of HIV [21].

Multiple mechanisms have been proposed by which GBV-C infection modulates HIV infection. GBV C infection results in reduced expression of the CCR5 and CXCR4 on CD4 + t Cells and this was independent of any effect of HAART and HIV viral load [22]. One possible molecular mechanism that plays the role in inhibiting the surface expression of CCR5 receptors is the binding of GBV-C E2 to CD81 on CCR5+ cells [23]. The GB Virus C E2 protein interferes with HIV-1 membrane fusion in a dose dependent pattern [24]. With HAART, it could have additive or synergistic effects. In Patients receiving HAART, median GBV-C RNA levels increased and decreased upon interruption of HAART thus suggesting a reciprocal relationship between GBV-C and HIV viral dynamics [25].

GBV-C virus also plays its role by influencing cytokine profile. It modulates the immune response. It is observed that Th2 cytokines levels are increased in HIV infected patients possibly due to Th2 polarization and Th1 cytokines are decreased. But in patients with concomitant infection, levels of Th1 cytokines are increased specially IL- 2 levels. Also it increases CD80+ plasmocytoid dendritic cells (pDCs) which produces Th1 cytokines. This further strengthens that GBV-C has multiple modes of action in reducing the HIV viral load [3,4].

This phenomenon of suppression of an infectious agent by another is also reported in other cases but on a small scale with relatively scarce data. This includes super infection of HIV infected patients with Dengue Virus of Flaviviridae and Measles virus of Paramyxoviridie [2,26].

The treatment and prevention protocols in current use advocate the use of pharmacological and surgical interventions for interrupting the course of disease process. Our hypothesis provides an alternative method to the existing treatment modalities in altering the course of disease.

## Speculating advantages of VIVI over HAART

HAART (Highly active antiretroviral therapy) has transformed HIV infection from a rapid, deadly disease into a chronic condition. However, the need to administer medicines for decades rather than years encourages us to think of another alternative.

The success of HAART therapy depends on patient adherence. For this purpose, interventional strategies with behavioral modification are required. Strategies includes motivation to begin therapy, then education with simultaneous involvement of patient in decision making, providing reliable access to primary medical care and medication. The concept of adherence is not limited to the patient but it includes clinician who develops a healthy and trustful physician-patient relationship [27,28]. Younger age, poor housing conditions, lack of social support, and problems of adherence with previous antiretroviral regimens were found to be the significant factors for non-adherence [29]. In order to overcome the issue of adherence, DOT-HAART (directly observed therapy with highly active antiretroviral therapy) was introduced in some countries. However meta-analysis of studies show no advantage of such practice over self-administered treatment [30]. The side effects of HAART therapy has led to many people discontinuing their therapy. This has been demonstrated by several studies [31,32].

Our treatment regimen offers solution to the problems of adherence and high financial burden associated with HAART. The use of VIVI method for treatment of HIV/AIDS provides us an alternative approach to treatment without the associated difficulties with patient compliance and the side effect profile of HAART drugs.

If our hypothesis is proven true, there are greater chances of fewer side effects or very rare effects like the vaccinations in current use which are nearly safe with adverse reactions ranging one in over million patients. Our therapy could possibly increase adherence of AIDS patients to HAART. Since some people stopped therapy due to therapy failure which was associated with the type of treatment or to the most recent HIV-RNA [32]. Combining our regiment with HAART may have some encouraging aspects through the additive effects as GBV-C has separate mechanisms through which it inhibits HIV replication. With on-going skepticism about the use of anti-retroviral agents for prophylaxis in HIV, VIVI can also be tested as a prophylactic intervention for primary prevention [33].

## Testing the hypothesis

Rigorous testing is required before converting this hypothesis into a mass scale treatment option for HIV patients. Science demands experiments to be conducted on animal models before their implementation on humans. However, a satisfactory animal model for GBV-C does not exist. Although GBV-C has been known to infect old-world primates, its propensity to infect new world primates remains somewhat controversial. Since chimpanzee experiments are no longer likely to be done, this makes the option of using an animal model for testing the hypothesis infeasible. Under these circumstances, it is plausible that humans with advanced HIV be used instead of animal models, since a significant number of people already receive licensed blood products that contain GBV-C.

Although in previous studies, cohorts have shown resolution of infection by another agent, none of them was deliberately infected with the virus. In our research model, three groups should be made of patients harboring multi-resistant strains of HIV. First is the Control group with standard treatment, second is the Case group A with superinfection while the third group is Case group B with both standard treatment and superinfection. They should be followed prospectively for a longer period. If the Case Group A or B shows improved primary outcome in terms of survival or secondary outcomes such as resolution of symptoms, i.e. Increase in CD4+ T cells, decrease in HIV RNA or proteins, increase in TH1 cytokines, decrease in opportunistic infections, our hypothesis will be confirmed.

## Implications of hypothesis

This hypothesis provides an opportunity to introduce novel methods in the treatment of chronic or unsuccessful diseases like HIV. Selection and usage of non-pathogenic strains means not having to deal with the side effects of pharmacological interventions. New vaccinations can be developed on basis of viral proteins. Also after developing a single working strain with maximum efficacy and lesser pathogenicity, we will be able to provide it at a more affordable cost to people with severe life threatening infections.

## Competing interests

The authors declare they have no conflict of interest. They did not receive any funding for the project.

## Authors' contributions

AS conceived the hypothesis and drafted the initial manuscript. MFK and MMN were involved in critical revision of the manuscript. All authors have read and approved the final manuscript.

## References

[B1] Xiang J Wünschmann S Diekema DJ Klinzman D Patrick KD George SL Stapleton JT Effect of coinfection with GB virus C on survival among patients with HIV infection N Engl J Med 2001 345 10 707 714 10.1056/NEJMoa003364 11547739

[B2] Watt G Kantipong P Jongsakul K Decrease in human immunodeficiency virus type 1 load during acute dengue fever Clin Infect Dis 2003 36 8 1067 10.1086/374600 12684921

[B3] Nunnari G Nigro L Palermo F Attanasio M Berger A Doerr HW Pomerantz RJ Cacopardo B Slower progression of HIV-1 infection in persons with GB virus C co-infection correlates with an intact T-helper 1 cytokine profile Ann Intern Med 2003 139 1 26 30 10.7326/0003-4819-139-1-200307010-00009 12834315

[B4] Lalle E Sacchi A Abbate I Vitale A Martini F D’Offizi G Antonucci G Castilletti C Poccia F Capobianchi M Activation of interferon response genes and of plasmacytoid dendritic cells in HIV-1 positive subjects with GB virus C co-infection Int J Immunopathol Pharmacol 2008 21 1 161 18336742 10.1177/039463200802100118

[B5] Leary TP Muerhoff S Simons JN Pilot Matias TJ Erker JC Chalmers ML Schalauder GG Dawson GJ Desai SM Mushahwar IK Sequence and genomic organization of GBV C: A novel member of the flaviviridae associated with human non A E hepatitis J Med Virol 1996 48 1 60 67 10.1002/(SICI)1096-9071(199601)48:1<60::AID-JMV10>3.0.CO;2-A 8825712

[B6] Stapleton JT GB virus type C/hepatitis G virus 2003 2003 New York: Thieme-Stratton, c1981 137 148

[B7] Alter MJ Gallagher M Morris TT Moyer LA Meeks EL Krawczynski K Kim JP Margolis HS Acute non-A–E hepatitis in the United States and the role of hepatitis G virus infection N Engl J Med 1997 336 11 741 746 10.1056/NEJM199703133361101 9052651

[B8] Alter HJ Nakatsuji Y Melpolder J Wages J Wesley R Shih JWK Kim JP The incidence of transfusion-associated hepatitis G virus infection and its relation to liver disease N Engl J Med 1997 336 11 747 754 10.1056/NEJM199703133361102 9052652

[B9] Xiang J McLinden JH Rydze RA Chang Q Kaufman TM Klinzman D Stapleton JT Viruses within the Flaviviridae decrease CD4 expression and inhibit HIV replication in human CD4+ cells J Immunol 2009 183 12 7860 7869 10.4049/jimmunol.0902276 19923460 PMC3557943

[B10] Ren FR Wang Y Li H Chen HS Zhao HY Hepatitis G virus infection in screened Chinese blood donors Vox Sang 1998 74 1 51 52 10.1046/j.1423-0410.1998.7410051.x 9481862

[B11] Tanaka Y Mizokami M Orito E Ohba K Nakano T Kato T Kondo Y Ding X Ueda R Sonoda S GB virus C/hepatitis G virus infection among Colombian native Indians Am J Trop Med Hyg 1998 59 3 462 467 9749645 10.4269/ajtmh.1998.59.462

[B12] Bassit L Kleter B Ribeiro-dos-Santos G Maertens G Sabino E Chamone D Quint W Saez-Alquezar A Hepatitis G virus: prevalence and sequence analysis in blood donors of São Paulo, Brazil Vox Sang 1998 74 2 83 87 10.1159/000030910 9501405

[B13] Dawson GJ Schlauder GG Pilot Matias TJ Thiele D Leary TP Murphy P Rosenblatt JE Simons JN Martinson FEA Gutierrez RA Prevalence studies of GB Virus C infection using reverse transcriptase polymerase chain reaction J Med Virol 1996 50 1 97 103 10.1002/(SICI)1096-9071(199609)50:1<97::AID-JMV16>3.0.CO;2-V 8890047

[B14] Hyland C Mison L Solomon N Cockerill J Wang L Hunt J Selvey L Faoagali J Cooksley W Young I Exposure to GB virus type C or hepatitis G virus in selected Australian adult and children populations Transfusion 1998 38 9 821 827 10.1046/j.1537-2995.1998.38998409001.x 9738621

[B15] Masuko K Mitsui T Iwano K Yamazaki C Okuda K Meguro T Murayama N Inoue T Tsuda F Okamoto H Infection with hepatitis GB virus C in patients on maintenance hemodialysis N Engl J Med 1996 334 23 1485 1491 10.1056/NEJM199606063342301 8618602

[B16] Thomas H Pickering J Karayiannis P Identification, prevalence and aspects of molecular biology of hepatitis G virus J Viral Hepat 1997 4 51 54 9097278 10.1111/j.1365-2893.1997.tb00160.x

[B17] Williams CF Klinzman D Yamashita TE Xiang J Polgreen PM Rinaldo C Liu C Phair J Margolick JB Zdunek D Persistent GB virus C infection and survival in HIV-infected men N Engl J Med 2004 350 10 981 990 10.1056/NEJMoa030107 14999110

[B18] Vahidnia F Petersen M Stapleton JT Rutherford GW Busch M Custer B Acquisition of GB virus type C and lower mortality in patients with advanced HIV disease Clin Infect Dis 2012 55 7 1012 1019 10.1093/cid/cis589 22752515 PMC3657520

[B19] Xiang J George SL Wunschmann S Chang Q Klinzman D Stapleton JT Inhibition of HIV-1 replication by GB virus C infection through increases in RANTES, MIP-1alpha, MIP-1beta, and SDF-1 Lancet 2004 363 9426 2040 2046 10.1016/S0140-6736(04)16453-2 15207954

[B20] Supapol WB Remis RS Raboud J Millson M Tappero J Kaul R Kulkarni P McConnell MS Mock PA Culnane M McNicholl J Roongpisuthipong A Chotpitayasunondh T Shaffer N Butera S Reduced mother-to-child transmission of HIV associated with infant but not maternal GB virus C infection J Infect Dis 2008 197 10 1369 1377 10.1086/587488 18419578

[B21] Giret MT Kallas EG GBV-C: state of the art and future prospects Curr HIV/AIDS Rep 2012 9 1 26 33 10.1007/s11904-011-0109-1 22246585

[B22] Schwarze-Zander C Neibecker M Othman S Tural C Clotet B Blackard JT Kupfer B Luechters G Chung RT Rockstroh JK GB Virus C (GBV-C) coinfection in advanced HIV disease is associated with low CCR5 and CXCR4 surface expression on CD4+ T cells Antivir Ther 2010 15 5 745 10.3851/IMP1602 20710056 PMC3210471

[B23] Nattermann J Nischalke HD Kupfer B Rockstroh J Hess L Sauerbruch T Spengler U Regulation of CC chemokine receptor 5 in hepatitis G virus infection AIDS 2003 17 10 1457 10.1097/00002030-200307040-00006 12824783

[B24] Herrera E Tenckhoff S Gómara MJ Galatola R Bleda MJ Gil C Ercilla G Gatell JM Tillmann HL Haro I Effect of synthetic peptides belonging to E2 envelope protein of GB virus C on human immunodeficiency virus type 1 infection J Med Chem 2010 53 16 6054 6063 10.1021/jm100452c 20718496

[B25] Björkman P Flamholc L Molnegren V Marshall A Güner N Widell A Enhanced and resumed GB virus C replication in HIV-1-infected individuals receiving HAART AIDS 2007 21 12 1641 10.1097/QAD.0b013e32823bc9b7 17630561

[B26] Moss WJ Ryon JJ Monze M Cutts F Quinn TC Griffin DE Suppression of human immunodeficiency virus replication during acute measles J Infect Dis 2002 185 8 1035 1042 10.1086/340027 11930312

[B27] Williams A Friedland G Adherence, compliance, and HAART AIDS Clin Care 1997 9 7 51 11364415

[B28] Nischal K Khopkar U Saple D Improving adherence to antiretroviral therapy Indian J Dermatol Venereol Leprol 2005 71 5 316 10.4103/0378-6323.16780 16394454

[B29] Spire B Duran S Souville M Leport C Raffi F Moatti JP Adherence to highly active antiretroviral therapies (HAART) in HIV-infected patients: from a predictive to a dynamic approach Soc Sci Med 2002 54 10 1481 1496 10.1016/S0277-9536(01)00125-3 12061483

[B30] Ford N Nachega JB Engel ME Mills EJ Directly observed antiretroviral therapy: a systematic review and meta-analysis of randomised clinical trials Lancet 2009 374 9707 2064 2071 10.1016/S0140-6736(09)61671-8 19954833

[B31] Bassetti S Battegay M Furrer H Rickenbach M Flepp M Kaiser L Telenti A Vernazza PL Bernasconi E Sudre P Why Is Highly Active Anti-retroviral Therapy (HAART) not prescribed or discontinued? J Acquir Immune Defic Syndr 1999 21 2 114 119 10360802

[B32] Monforte AA Lepri AC Rezza G Pezzotti P Antinori A Phillips AN Angarano G Colangeli V Luca AD Ippolito G Caggese L Soscia F Filice G Gritti F Narciso P Tirelli U Moroni M Insights into the reasons for discontinuation of the first highly active antiretroviral therapy (HAART) regimen in a cohort of antiretroviral naive patients AIDS 2000 14 5 499 507 10.1097/00002030-200003310-00005 10780712

[B33] Anwer SMS Ansari MH Clouds with silver lining: Defining conditions that serve as blessings in disguise El Mednifico Journal 2012 1 1 19

